# Containing misinformation: Modeling spatial games of fake news

**DOI:** 10.1093/pnasnexus/pgae090

**Published:** 2024-02-27

**Authors:** Matthew I Jones, Scott D Pauls, Feng Fu

**Affiliations:** Sociology Department, Yale University, New Haven, CT 06511, USA; Mathematics Department, Dartmouth College, Hanover, NH 03755, USA; Mathematics Department, Dartmouth College, Hanover, NH 03755, USA; Mathematics Department, Dartmouth College, Hanover, NH 03755, USA; Department of Biomedical Data Science, Dartmouth College, Hanover, NH 03756, USA

**Keywords:** social networks, online misinformation, fact-checking, echo chambers, spatial game theory

## Abstract

The spread of fake news on social media is a pressing issue. Here, we develop a mathematical model on social networks in which news sharing is modeled as a coordination game. We use this model to study the effect of adding designated individuals who sanction fake news sharers (representing, for example, correction of false claims or public shaming of those who share such claims). By simulating our model on synthetic square lattices and small-world networks, we demonstrate that social network structure allows fake news spreaders to form echo chambers and more than doubles fake news’ resistance to distributed sanctioning efforts. We confirm our results are robust to a wide range of coordination and sanctioning payoff parameters as well as initial conditions. Using a Twitter network dataset, we show that sanctioners can help contain fake news when placed strategically. Furthermore, we analytically determine the conditions required for peer sanctioning to be effective, including prevalence and enforcement levels. Our findings have implications for developing mitigation strategies to control misinformation and preserve the integrity of public discourse.

**Significance Statement**

Modern social media has been inundated by false and misleading headlines and articles. We advance the study of such fake news by developing a game-theoretic model of the spread of fake news in a social network. Our model reveals that the structure of a social network can limit the efficacy of sanctioning efforts by a factor of two to three. However, we find that by strategically selecting fake news sanctioners using network-based methods, the efficacy of fact-checking can be significantly improved in certain networks.

## Introduction

The proliferation of fake news on social media has touched many aspects of society, from influencing terrorist attacks (1–3) to the COVID-19 pandemic (4–7) to politics and elections (8–12). There is widespread concern that social media platforms have become an effective mechanism for rapidly spreading false information by privileging attention-grabbing headlines over a nuanced understanding of complex topics (13–15).

A great deal of scholarship has gone into studying different methods of limiting misinformation, the majority of which focuses on the actions of centralized authorities like the social media companies. Potential interventions include altering the social media landscape (by adding warnings (16, 17) or attention checks (18)) or participants (by increasing media literacy (19) or “innoculating” against fake news (20)). A smaller subset of the field has examined the role individual users can play in suppressing fake news by correcting misinformation whenever they see it (21, 22). This paper builds on such decentralized fact-checking work by studying the problem through theoretical modeling.

Recent studies have sought to understand the mechanisms by which false stories gain traction and reach wide audiences despite containing blatant falsehoods. Shin et al. (14) examined the lifecycle of 17 popular political rumors on Twitter during the 2012 US presidential election; they found that misinformation tends to come back multiple times after the initial publication, while facts do not. Using massive Twitter datasets, Vosoughi et al. (15) reported that the spread of true and false news follow distinctive patterns: falsehood diffused faster, deeper, and more broadly than the truth in all categories of information. These studies and others suggest that fake news has some innate advantage over real news when shared on online platforms.

Making matters worse, social influence, following, and unfollowing on online social networks such as Twitter can lead to the emergence of polarized and segregated structures commonly referred to as *echo chambers* which create the necessary conditions for confirmation bias and selection bias (23). Evans and Fu (24) investigated opinion formation on dynamic social networks and, using the voting records of the United States House of Representatives, presented and validated the conditions for the emergence of partisan echo chambers (25–27). More recently, Wang et al. integrated publicly available Twitter data with an agent-based model of opinion formation driven by sociocognitive biases and demonstrated that the open-mindedness of individuals is a key determinant in forming echo chambers under dueling campaign influence (28).

Here, we explore the impact of individual social media users taking action against fake news sharers. To do so, we develop a mathematical model based on spatial game theory. This work uses spatial games to study problems of coordination and collective action, as well as previous research that has found network structure can reinforce good behavior (29, 30). The evolution of these systems can exhibit interesting spatial phenomenon that is not present in the well-mixed case (31), so this is an ideal model to use to study echo chambers in an online social network.

Recent research in opinion dynamics on networks has focused on understanding the conditions under which consensus of opinion may emerge in a population, as well as those that cause divergence of opinion and weaken information transfer (32–38). In our work, there are two narratives, the true narrative and a false news narrative. A successful outcome for our model is population-wide consensus on the true narrative, but we frequently see a middle ground where isolated communities form echo chambers and continue to hold minority beliefs.

Within this framework, we study the effect of individuals who choose to sanction users who share fake news. There is good evidence to suggest that user corrections can be effective at correcting false information in pairwise interactions (21, 22), perhaps because such corrections serve as attention cues (39) and perhaps because they are a punishment that harms the reputation of the fake news sharers (40, 41). Inspired by “zealot models” from the field of opinion dynamics (37), we assume these fake news punishers are highly motivated and therefore immune to the social pressure of their neighbors to consume fake news. Alternatively, with the rise of artificial intelligence, this simulates embedding large language model-equipped fact-checker bots into a network (42, 43).

In sum, we present an agent-based model of distributed sanctioning of fake news sharers in spatial game. We quantify the density of sanctioners in the population and study how agents share real or fake news depending on the behavior and success of their neighbors. Our model is analyzed with simulations as well as rigorous mathematical analysis, and we find that echo chamber structures impede crowdsourced sanctioning, thereby requiring significantly higher levels of sanctioners to successfully contain online misinformation.

## Methods and model

This paper presents a model of fake news propagation that is designed to capture the most important properties of the virtual interactions that occur all the time on online social media sites as misinformation is being shared. Suppose there are two competing narratives, one factual (called *A*) and one false (called *B*), that are spreading on social media as groups of supporters choose to go along with a narrative by sharing/interacting with posts. There are many examples of this, including vaccine safety (7), election fraud (8), and school shootings (2). In a typical interaction, an individual makes a post which is then seen by all of her followers; like-minded followers may interact positively with this post by liking or sharing, while those in the other group may simply ignore the post or even attempt to debunk a false story by pointing out flaws, sharing a link to a fact-checking website, or even flagging the post as false.

We also want to take into account that these interactions happen on social networks with limited connectivity, not in an open space where everyone knows everyone and sees everything. We use a network where vertices represent individuals that can exhibit three distinct behaviors: supporting the factual *A* narrative, supporting the false *B* narrative, and sanctioning people who share the false narrative (*C*). Individuals will receive a payoff depending on their behavior and the behavior of their neighbors. These payoffs are encoded in the payoff matrix like the one below:


(1) $$\begin{array}{c} \begin{array}{cccc} & A & B & C \end{array}\\ \begin{array}{c} A\\ B\\ C \end{array}\left(\begin{array}{ccc} 1 & 0 & 1\\ 0 & 2 & -4\\ 0 & 0 & 0 \end{array}\right). \end{array}$$

To read a payoff matrix, look at the row corresponding to an individual’s strategy and the column corresponding to her neighbor’s strategy. For example, when an *A* player interacts with a *C* player, the *A* player gets a payoff of 1 and the *C* player gets a payoff of 0.

These payoffs can be thought of as the average value gained from being an *A*, *B*, or *C* player across many interactions over time. In our model, real and fake news sharers both get positive payoffs when interacting with like-minded individuals, which represents the social capital gained through online engagement, such as likes, shares, retweets, and comments. However, our payoff matrix provides a larger intrinsic benefit to the *B* (false) narrative. There are many reasons why these groups have different coordination effects. Fake news, particularly viral posts, seem to spread better than real news according to almost every metric (14, 15). Additionally, fake news is often very extreme (see Refs. (1, 2) for troubling examples) and elicits strong reactions in an online environment. Accordingly, we see that more extreme individuals tend to follow elites who share more false information (11). Finally, social media algorithms may be incentivized to boost the visibility of fake news that is driving user interactions and therefore revenue (44). All these factors result in fake news being more likely to shared or retweeted than real news, so we compress all these different effects into a single higher payoff for *B*–*B* interactions compared to *A*–*A* interactions. However, this is not strictly necessary for our main result showing that network structure suppresses distributed sanctioning. In the supplementary material, we test the effects of our parameter choices by looking at the critical sanctioner density needed to give real news the advantage for a wide range of values of both the *B*–*B* payoff and the *B*–*C* punishment terms. We see that if fake news has any natural advantage over real news, the structure of the network requires an addition 10–20% of the population sanctioning fake news. This also holds for a wide range of punishment factors.

To contain the spread of fake news, this natural advantage given to the *B* narrative will have to be counterbalanced by a penalty inflicted when meeting *C* players. Because these are highly motivated individuals, they behave like supporters of the *A* narrative when they see *A* posts online, but they are also willing to publicly refute fake news when encountered on social media. The effect of sanctioning can be felt in three ways. First, a fact-check can directly correct the false information, which is a social punishment for the *B* player and makes posting about the *B* narrative less appealing. Second, this correction is an attention check and a source for more accurate information so it prevents others from repeating the false information. Finally, they can flag the posts as misinformation, enabling the social media algorithm to label the post as false or even suppress the post’s visibility. In all cases, the fitness of the *B* narrative is decreased as its desirability, credibility, and visibility are lowered. Because of their high analytical reasoning abilities (or the fact that they are bots), our sanctioners will never change strategy, playing *C* during every time step. Therefore, the proportion of sanctioners $p_{C}$ is prescribed and static. Because of this, the payoff to sanctioners is irrelevant, so for simplicity we set it to zero. Of course, it is inevitable that crowdsourced fact-checking will result in errors. Either through individual biases, incorrect information elsewhere, or technical problems, sanctioners may occasionally punish accurate news or endorse misinformation. We consider this possibility in SI Section 2.

A selection strength parameter controls how much impact an individual’s payoff has on her reproductive success in the update step. The payoffs and selection strength can take arbitrary numerical values, but for the rest of this paper, unless otherwise noted, we will use a selection strength of β=0.5 and the payoff matrix for this symmetric, two-player game will be the matrix in Eq. 1.

Thirty-five percent of tweets are retweets and another 40% are replies (45), meaning a significant fraction of online activity is essentially imitation. Additionally, individuals who see that only certain types of stories are receiving positive feedback may become convinced of the accuracy of those (potentially false) narratives (46) and begin sharing those same stories themselves. To capture these social imitation phenomena, we will employ a death–birth process for the evolutionary strategy update (29) of our model. After computing the expected payoff $\pi_{i}$ for every individual *i*, a focal individual imitates the strategy of one of her neighbors, chosen with probability proportional to their fitness $f_{i}=\text{exp}(\;\beta \pi_{i})$. Thus, individuals with high payoff are likely to be selected. This captures individuals who “change sides” because they see that posts from one narrative are generating lots of positive feedback, but it also models those individuals who are only exposed to one of the two narratives and therefore tricked (in some sense) into going along with it due to inattentiveness (39).

In our investigation, we utilize two variants of the update rule: synchronous and asynchronous. In the synchronous update, used in our simulations, every individual updates their strategy simultaneously. Conversely, in the asynchronous update, which lends itself to easier mathematical analysis, a single individual is chosen uniformly at random to update. These two update rules will lead to very similar outcomes, and the minor differences between them are manifested only in edge cases that occur rarely for reasonable sanctioner densities. Keeping this in mind, we will treat them as qualitatively identical processes operating on different time scales.

**Fig. 1.**
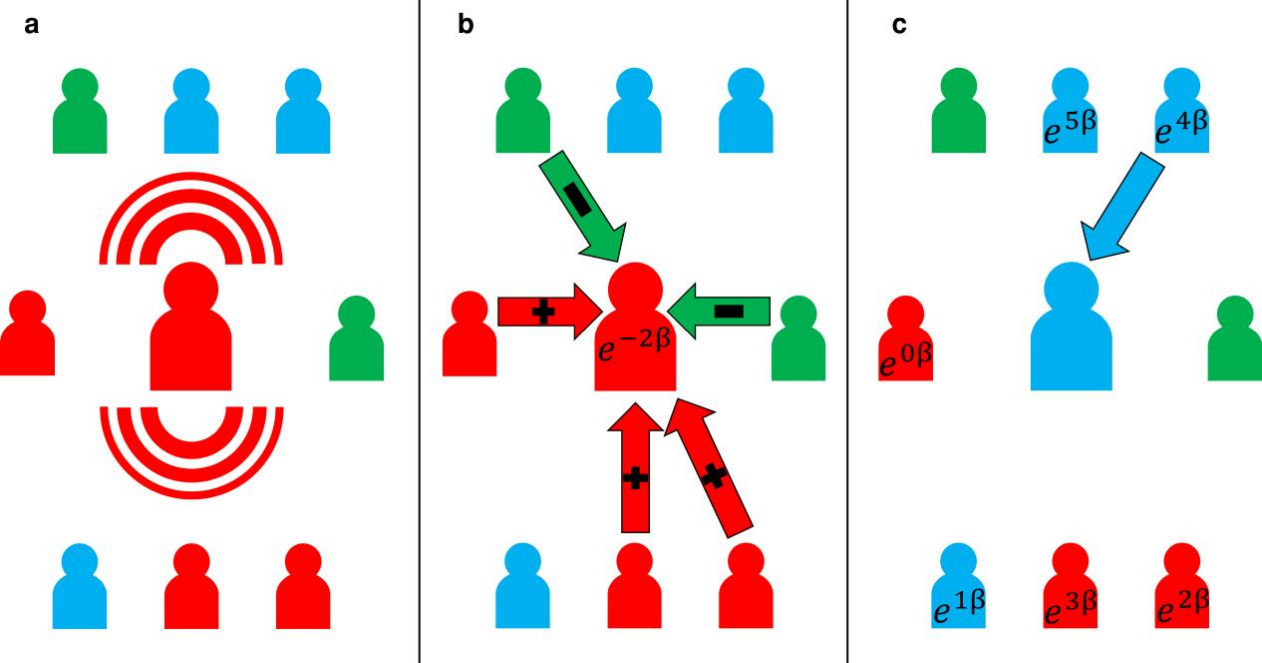
Model schematic. We model information sharing and sanctioning through the lens of spatial games. First, individuals share news that is either true or false, as illustrated in a). In b), a focal individual receiving positive or negative feedback from her neighbors depending on their relative beliefs. The presence of crowdsourced sanctioners can significantly reduce the fitness of fake news sharers. Lastly, in c), individuals update their strategy by copying a neighbor proportional to fitness. However, sanctioners do not update strategy and are never chosen to be replicated.

The basic outline of our model is shown in Fig. 1. First, individuals play the fake news game with neighbors by broadcasting a post aligning with the *A* or *B* narrative. The expected payoff from these games is then converted into a fitness measure. Figure 1c demonstrates the asynchronous update, where only a single focal individual updates strategy by considering the fitness of all her neighbors. In the synchronous update, all individuals would select a neighbor simultaneously.

Our study of the spread of fake news focuses on three distinct network topologies with unique properties: a 30×30 square lattice (31), Watts–Strogatz small-world networks (47) (also with n=900), and a portion of the Twitter follower network (48) (n=404,719). Our small-world networks are calibrated to have the desired high clustering coefficients and short path lengths using the following parameters: base degree 8 and rewiring probability 0.03, giving us approximately 200 shortcuts. The Twitter network is interesting for its size but also its natural clustering and the gatekeeping individuals that control the flow of information through the network. Although edges in the network were originally directed, we symmetrized the network before using it to match the bidirectional flow of information in our model.

To initialize our simulations, we assign some fraction $p_{C}$ of the individuals as sanctioners, and the rest we set to be *A* or *B* players with equal probability. This gives us a strong fake news presence throughout the network and therefore a strong signal from sanctioning. However, the observed prevalence of fake news spreaders in certain populations is much lower (49), so we also run the model on the full range of initial distributions of *A* and *B* players. These results can be found in the supplementary material. Network structure has a strong impact on how the initial distribution affects population dynamics. Small-world and lattice networks respond very differently as the ratio of *A* and *B* players changes.

After initializing, we allow the system to evolve using one of the update processes described above until all possible players are sharing the same type of news or a predetermined number of time steps is reached. At the end of the simulation, the type of news with more sharers is said to be dominant. If there are no individuals sharing one type of news, we say that that strategy has gone extinct and the other strategy has fixated.

## Results

We used both computer simulations and analytic techniques to study this spatial game of fake news. Our simulations demonstrate that the spontaneous formation of echo chambers can be driven by local variation in sanctioner density, and that these echo chambers are extremely resistant to invasion. We also test the hypothesis that network structure seems to protect fake news from sanctioning, and we examine the viability of targeted inoculation strategies in which sanctioners are carefully selected to maximize their impact with minimal resources. Finally, we use analytic techniques to determine which collections of payoff values favor invasion by real news, fake news, or neither.

### Echo chamber formation

When there are very few sanctioners, the natural advantage that fake news sharers have allows them to drive the real news sharing strategy to extinction. Similarly, when there is a sufficient sanctioner presence, the risk of punishment for spreading misinformation is too great and the entire population eventually converges to sharing real news. However, there is a wide range of sanctioner densities where neither strategy is quickly driven to extinction and instead we see the spontaneous formation of echo chambers in our simulations. These echo chambers emerge without any deliberate seeding from a noisy initial state where real and fake news sharers each make up approximately 50% of the population. We do not assume the homophily in these structures; instead, they form because of the self-sorting process intrinsic to many social media platforms fueled by social imitation.

**Fig. 2.**
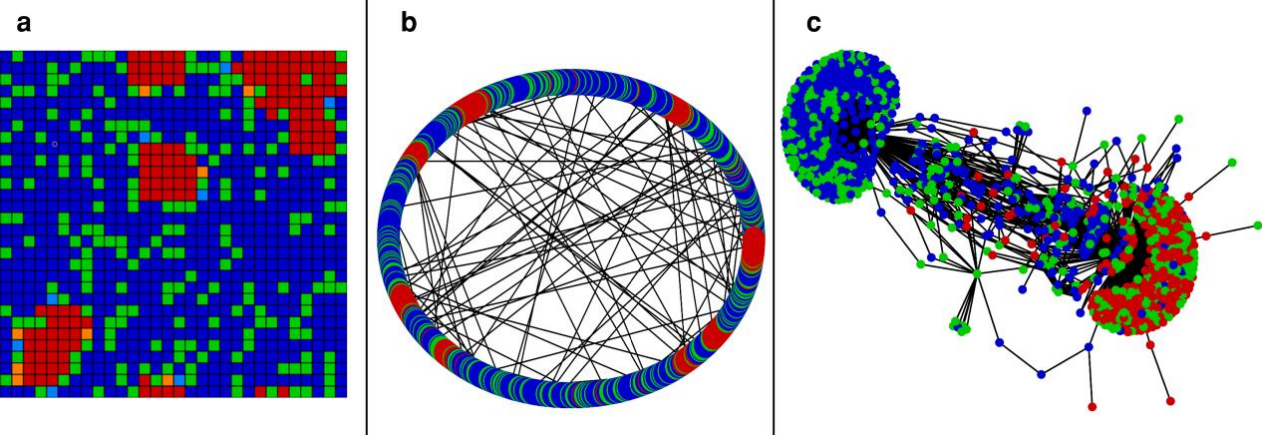
Echo chambers of fake news spreaders in a majority real news-spreading population that are isolated from the rest of the population. In a), the lightly shaded squares represent individuals who have recently changed strategy. The network in b) is a Watts–Strogatz small-world network, and c) is a small breadth-first subgraph of the Twitter network containing approximately 1,000 vertices. However, the simulation was run on the entire ≈400,000 vertex network (see Methods and model).

We define echo chambers by their longevity, as either real or fake news goes extinct unless the minority strategy manages to form small, highly interconnected communities that are secure from invasion by the majority strategy. For a more detailed analysis of the stability of these pseudosteady states, see the supplementary material. Figure 2 shows examples of these echo chambers on the three different network topologies we studied.

These echo chambers, once formed, are incredibly resistant to invasion, resulting in a *pseudosteady state* that cannot last forever, but will take an extremely long time to break down. After forming in relatively few time steps ( <100), these echo chambers remained largely unchanged for over 1 million time steps in our longest simulations. There are small variations in the pseudosteady state when specific individuals change behavior, but as a whole the echo chamber remains unchanged. Observe in Fig. 2a that the only individuals changing strategy are on the borders of the echo chambers in the system. Individuals on the periphery of the echo chamber are exposed to both real and fake news and may change strategy occasionally, but those in the interior are surrounded by like-minded individuals and have high fitness, which allows them to reinforce minority behavior by the more exposed peripheral individuals. Thus, it is very unlikely that a small perturbation on the border will result in any change to the interior of the echo chamber.

Our comprehensive simulations confirm that the formation of echo chambers occurs across a wide range of payoff values and selection strengths. Local variation in sanctioner density means in some areas there are no sanctioners, leaving room for a fake news echo chamber. In other areas, they form a protective wall that gradually becomes more difficult for fake news to penetrate as selection strength grows.

### Critical sanctioner density

**Fig. 3.**
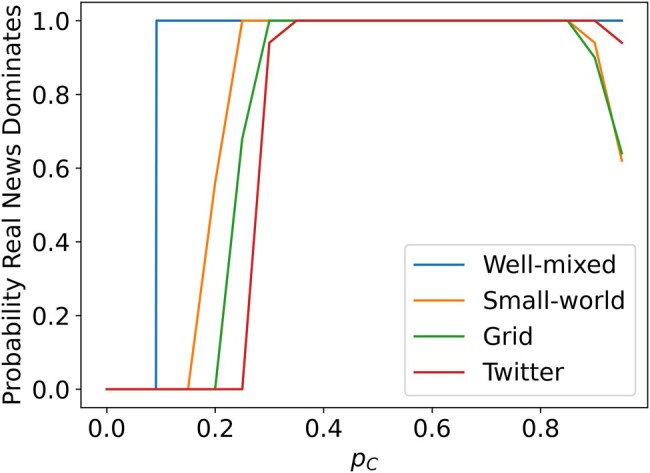
The probability that over half the viable population ends up sharing real news as a function of sanctioner density for different network topologies. The well-mixed result comes from analysis of replicator dynamics, and the rest are obtained from simulations. For very high values of $p_{C}$, the spreader layer breaks apart into isolated individuals and the dominant strategy is determined more by the random initialization than selection. The simulations consisted of 50 populations at 20 evenly spaced sanctioner densities. At 5,000 time steps, a pseudosteady state was declared and the simulation ended, except the Twitter simulations which ended at 500 time steps for computational reasons.

These echo chambers can be made up of fake news sharers, as in Fig. 2, or real news sharers depending on the density of sanctioners. The critical sanctioner density is the tipping point at which real news sharers are more likely to be in the majority than in the minority. Figure 3 shows how the probability that real news becomes the dominant strategy changes as the fraction of sanctioners increases. It is clear that the critical sanctioner density varies depending on the topology of the social network: $p_{c}\approx 0.235$ on the square lattice, $p_{C}\approx 0.2$ for small worlds, and $p_{C}\approx 0.275$ for the Twitter network.

We can compare these results to the simple case of an infinite, well-mixed population evolving according to replicator dynamics (50). In this case, we begin with a fraction $p_{C}$ of sanctioners and the remaining population evenly divided between real and fake news sharers ( $p_{A}=p_{B}=\frac{1-p_{C}}{2}$), and we consider the relative payoffs of *A* and *B* players when choosing a random opponent under the payoff matrix [1].

The expected payoff for an *A* player at t=0 is


(2) $$f_{A}(0)=1(p_{A})+1(p_{C})=\frac{1-p_{C}}{2}+p_{C}=\frac{1+p_{C}}{2}$$

and the expected payoff for a *B* player at t=0 is


(3) $$f_{B}(0)=2(p_{B})-4(p_{C})=2\frac{1-p_{C}}{2}-4p_{C}=1-5p_{C}.$$

Because this is a coordination game, if *A* has a higher initial fitness, the proportion of *A* players will grow and $f_{A}(t)$ will get only get larger while $f_{B}(t)$ gets smaller, until *B* becomes functionally extinct. Therefore, the fixation of *A* is favored over *B* if $f_{A}(0)>f_{B}(0)$, which can be solving for $p_{C}$ using the equations above. We get the critical threshold for $p_{C}$ is found to be


(4) $$p_{C}>\frac{1}{11}\approx 0.091.$$

We conclude that the network structure of the spatial game makes containing fake news significantly more challenging. In fact, between two to three times as many sanctioners are needed to contain the sharing of fake news in small, isolated echo chambers compared to a well-mixed population. Furthermore, additional sanctioners are needed to have a good chance of driving fake news sharing behavior to total extinction.

It is worth noting that for very high values of $p_{C}$, the probability that real news dominates actually decreases. This seemingly paradoxical result can be explained by noting that for such high values of $p_{C}$, the population of real and fake news sharers has completely broken down into small, disconnected components due to the large number of static sanctioners. These components typically have only one or two individuals and are therefore completely constrained by their initial conditions, leaving no opportunity for beneficial strategies to spread through selection. Fortunately, when selecting sanctioners randomly, this only occurs for unrealistically high values of $p_{C}$.

### Targeted sanctioning

**Fig. 4.**
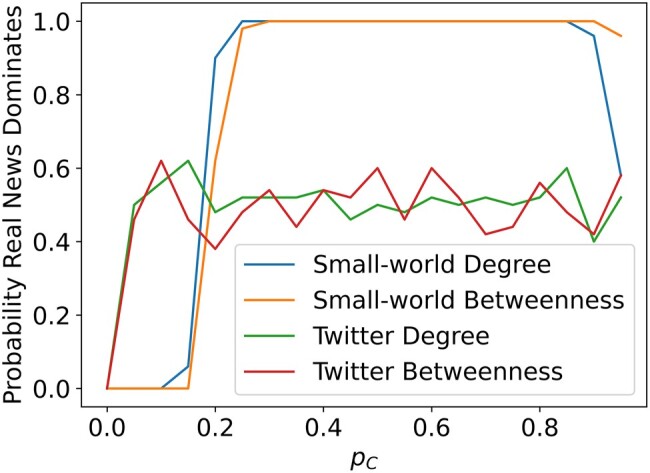
The probability of real news dominating on small-world networks and the Twitter network using the degree and betweenness centralities to place sanctioners. Once again, we run simulations with 50 iterations, 20 density values, and a limit of 5,000 (or 500) time steps.

So far, we have only considered populations where sanctioners are placed randomly. However, in almost all networks, certain vertices are more centrally located than others, and this effect is particularly pronounced in naturally formed social networks. To improve the efficiency of crowdsourced sanctioning with limited resources, it is vitally important to study targeted intervention algorithms by selecting the individuals who will have the most impact. Our findings, shown in Fig. 4, focus on two measures of network centrality, degree (the number of edges attached to a vertex) and betweenness (51). Intuitively, selecting vertices with the largest degree will maximize the number of chances sanctioners will have to punish fake news, because they will play more games against more opponents than the vertices with low degree. Betweenness centrality, on the other hand, will be selecting vertices that are most critical to transferring information between vertices. Thus, selecting by betweenness centrality could theoretically remove important pathways fake news needs to spread from one part of the population to another. However, there are many more centrality measures and the problem of selecting individuals for optimal sanctioning remains an open problem. Since all vertices in an infinite square lattice have the same centrality, our work here is restricted to small-world networks and the Twitter network.

Figure 4 has several interesting features. First, we see that in small worlds, using the degree and betweenness centralities have virtually the same performance. This is expected as the additional shortcut edges in small worlds create short path lengths, resulting in individuals with high degree also having high betweenness centrality. More surprising is the fact that targeted sanctioning is only marginally more successful than random placement, as seen by comparing Figs. 3 and 4. This may be due to the relatively uniform nature of small-world networks, where there is little variation from vertex to vertex.

In contrast, the Twitter network, with its diverse degree distribution, exhibits a substantial change in the effectiveness of targeted vs. random sanctioning. By targeting high degree or betweenness centrality individuals to be sanctioners, we quickly separate the real and fake news sharers (*A* and *B*) in the network into disconnected singletons and pairs, as these networks become disconnected very quickly when high degree vertices are removed from the network (52). Therefore, it is about equally likely that the initial random distribution will have more fake or real news sharers, so the probability that real news “dominates” by being present in over half the viable population hovers around 0.5 for almost all values of sanctioner density. We observed a similar effect for high values of $p_{C}$ in Fig. 3.

As shown in Fig. 4, on the Twitter network, this happens almost immediately. A very small percentage of sanctioners (<5%) is needed to break the paths of information transfer that fake news needs to spread. This suggests that in real-world networks, a targeted crowdsourced sanctioning effort where sanctioners are **also** encouraged to share real news with their neighbors could be highly effective with relatively little collective effort. In this scenario, the network structure will actually benefit real news instead of fake news by removing important vertices that fake news needs to move through to get to the rest of the population, while still allowing real news to spread. To further enhance our model, we plan to explore the effectiveness of allowing sanctioners to share real news while still stopping fake news, as this may improve the performance of targeted sanctioning algorithms.

### Analytic results under weak selection

The selection strength *β* determines the effect payoff from the fake news game has on reproductive success. As *β* approaches zero, the evolution of the system comes to resemble *neutral drift*, in which individuals choose strategy with no regard for payoff (29, 53). In this domain, the pseudosteady state with its echo chambers becomes short-lived, and the system quickly converges to all possible individuals sharing the same type of news. In the following section, we derive analytical results in this limit of weak selection.

Assuming a *k*-regular network structure like the square lattice, we will use an extended pair approximation method (54) to study the emergence and spread of real and fake news. In this section, we derive a closed-form expression for the fixation probability of *A*, the probability that a population with some initial condition evolves so that the entire viable population eventually evolves to play *A*. Our objective is to study the effects of changing the payoffs for real news, fake news, and sanctioners, so we will begin with a general payoff matrix:


(5) $$\begin{array}{c} \begin{array}{cccc} & A & B & C \end{array}\\ \begin{array}{c} A\\ B\\ C \end{array}\left(\begin{array}{ccc} a & b & \alpha \\ c & d & \gamma \\ 0 & 0 & 0 \end{array}\right) \end{array}.$$

In the limit of weak selection β≪1, we will obtain conditions for the fixation probabilities of *A* and *B* as functions of these payoff values.

When we suppose that we begin with a fraction *p* of *A* individuals, we can calculate the expected value $m_{A}(p)$ and variance $v_{A}(p)$ of the change in abundance of *A* during the asynchronous update step where a single random individual considers changing strategy. The fixation probability of *A* for an initial fraction *p* of *A* players, denoted by $\rho_{A}(p)$, satisfies the diffusion approximation equation for large populations (see Ref. (29) for details):


(6) $$m_{A}(p)\frac{d}{dp}\rho_{A}(p)+\left(\frac{v_{A}(p)}{2}\right)\frac{d^{2}}{dp^{2}}\rho_{A}(p)=0$$

with the boundary conditions $\rho_{A}(0)=0$ and $\rho_{A}(1)=1$. This equation has a closed-form solution, and thus we can obtain an exact formula for $\rho_{A}$.

Our derivation of the following explicit expressions for the fixation probabilities in terms of the payoff values, lattice degree *k*, and sanctioner density $p_{c}$ is detailed in the supplementary material. The final result is that, for small values of *p*,


(7) $$\rho_{A}(p)\approx p+\frac{\beta Np(1-p)}{6k}(-u_{1}-3u_{2}),$$

(8) $$\rho_{B}(p)\approx p+\frac{\beta Np(1-p)}{6k}(-w_{1}-3w_{2}),$$

where $u_{1}=(a-b-c+d)(1-k^{2}-\frac{1+k}{\left(p_{C}-1\right)})(1-p_{C})$, $u_{2}=-a+b+c-$  d−  $ak+bk-bk^{2}+dk^{2}+(k-1)(c+(b-\alpha +\gamma )k-d(1+k))p_{C}$, $w_{1}=u_{1}$, and $w_{2}=-(u_{1}+u_{2})$.

**Fig. 5.**
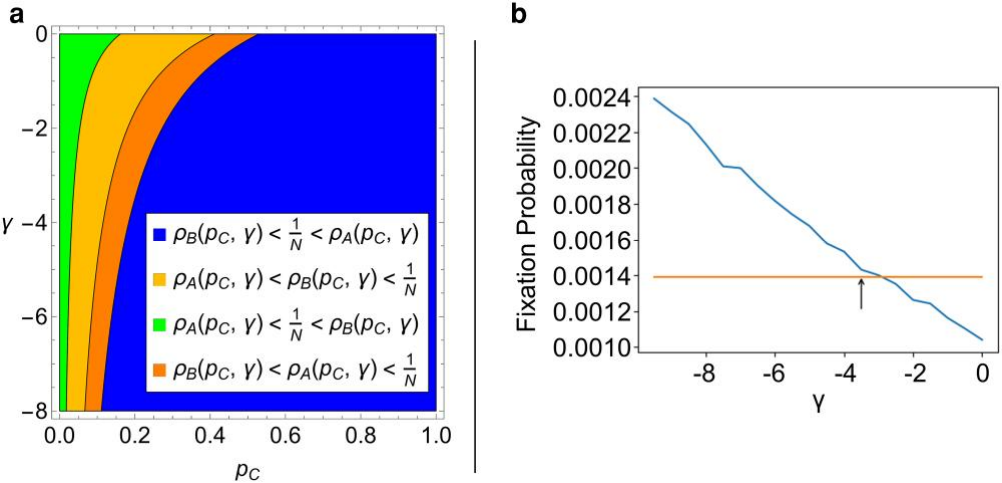
The fixation probabilities of real and fake news spreaders in the limit of weak selection using payoff values from (1), except for *γ* which varies from 0 to −8. In a), we see what regions of the $p_{C}-\gamma$ plane give true stories an advantage (far right region), fake news an advantage (far left region), or neither (middle regions). In b), we see an approximation of the fixation probability for a single real news sharer from simulations, when $p_{C}=0.2$ and β=0.0001. These simulation results intersect the threshold line $\frac{1}{N}\approx 0.0014$ close to where it was predicted by the analytic results, indicated by the arrow.

In particular, we may be interested in the emergence of new behavior in a previously homogeneous population. We calculate the fixation probability when beginning with a single initial *A* player, denoted by $\rho_{A}$, and derive the conditions for truthful behavior to be favored, that is, when $\rho_{A}>1/N$, where *N* is the size of the population. We also repeat the process for a single *B* player. Using Eqs. 7 and 8, we show the impact of $p_{C}$ and *γ*, the punishment defectors suffer from sanctioners, on the fixation probabilities of real and fake news in Fig. 5a.

This allows us to determine the conditions necessary for sanctioning to be effectively combat misinformation and quantify how high the penalty *γ* needs to be for a given proportion of sanctioners, $p_{C}$, in the system. In Fig. 5a, we see that for strong penalties ( γ<−4) only a fifth of the population or less needs to be sanctioners for selection to favor real news. However, if sanctioners are less willing to publicly shame fake news spreaders and *γ* gets closer to zero, the number of sanctioners need increase to about half the population. The green region of the $p_{C}-\gamma$ plane shows where selection favors fake news; this only happens when there are very few sanctioners. Notice that there is a wide region in orange where selection does not favor invasion by real or fake news. This is because the fake news game is a coordination game that tends to put minorities (like a single invading mutant) at a disadvantage. These analytic approximations closely match simulation testing, as shown in Fig. 5b.

## Discussion and conclusion

This work adds to the growing body of research surrounding fake news, echo chambers, and punishment and we believe that it has immediate implications for the study of misinformation. Our findings indicate that the spatial structure of social networks tends to hinder the sanctioning efforts of individuals inside the network, but by carefully selecting sanctioners, that same structure can be leveraged to combat misinformation by amplifying sanctioning efforts where they are most needed.

This paper contributes theoretical developments to a field that is predominantly empirical, but there are other model-based papers that have been published. Bak-Coleman et al. used data to fit a statistical model from infectious diseases, from which they drew several conclusions about the use of various interventions including fact-checking, nudges, and banning accounts (55). Several other papers have developed game-theoretic models to study the relationship between consumers and producers (44), the relationship between fake news and homophily (56), and (mis)information cascades on social networks (57). However, these game-theoretic models are primarily focused on characterizing the actions of individuals; in this paper, we took a wider look at population-wide dynamics (the rise and fall of sharing behaviors regarding fake vs. true news) as influenced by the presence of dedicated sanctioners.

Our analytic results allow us to easily test potential combinations of reward and punishment and use both “carrots and sticks” to encourage real news and dampen fake news. Like previous work studying public goods games, we see that a strong punishment of defectors is effective at stopping bad behavior (58–60).

Future work combining potential experimental behavior data (46) with our present model will help incorporate relevant social network and psychological factors in our research. In particular, the constants in the payoff matrix and the selection strength were chosen fairly arbitrarily (see the supplementary material for an investigation of some of these parameters). Analyzing real-world data may allow us better estimates of some of these values, which in turn can give better actionable advice about how to control the spread of fake news. Empirical studies can also confirm our predictions regarding the impact of rewards and punishments for sharing real and fake news on the ability of fake news to spread through a population.

There are two competing theories about the formation of echo chambers: one side suggests that social connections drives similarity of belief (46, 61), while the other side claims the opposite, that similar beliefs lead to the formation of social ties (62). Our work here shows that the spatial distribution of sanctioners can also contribute to echo chamber creation, but this work only represents the first steps toward understanding how localized policing of fake news impacts echo chamber formation. The formation of echo chambers is dependent on various factors, including selection strength, and there is much we still do not understand. Preliminary results show that the formation of resilient echo chambers is dependent on the topology of the social network, and while social media sites do resemble lattices or small worlds in some respects, there are other properties of social networks that may be more or less conducive to echo chamber formation.

Extensions of our present work on targeted sanctioning efforts will likely lead to useful insights for optimizing field deployment of crowdsourcing sanctioning. There will be a good deal of further work to do, for example, on using other network topologies and other targeting centralities. In addition, the use of larger network datasets will give us more realistic behavior as there may be large-scale social network features essential to the development of echo chambers that are not captured in any of the network models we used.

Finally, it is important to note that our work opens up new avenues for future research, such as extending targeting algorithms to multiplex networks that account for the interconnected ecosystems of social media platforms and multichannel communication. Incorporating social psychological factors such as heterogeneity of social influence, repeated exposure, and preexisting beliefs into these models will allow for a more comprehensive understanding of the spread of fake news and the effectiveness of sanctioning efforts. We believe that this research will not only provide a deeper understanding of the complexities of social network dynamics but also help inform practical interventions aimed at combating the spread of misinformation in the digital age.

## Supplementary Material

pgae090_Supplementary_Data

## Data Availability

The code for this article is publically available on Github at https://github.com/MattJonesMath/FakeNews.

## Previous Presentation

These results were previously presented at eSMB Annual Meeting 2020, SIAM Annual Meeting 2020, and $IC^{2}S^{2}$ 2020.

## Preprints

A preprint of this article is published at https://doi.org/10.48550/arXiv.2206.04118.
