# Supplementary material for: Containing misinformation: Modeling spatial games of fake news
Source: PNAS Nexus. 2024 Feb 27;3(3):pgae090. doi: 10.1093/pnasnexus/pgae090 (PMC10924450; doi:10.1093/pnasnexus/pgae090)
Supplement: pgae090_Supplementary_Data [file pgae090_supplementary_data.zip › PNASNEXUS-PNASNEXUS-2023-01200R-s02.pdf]

Containing Misinformation: Modeling Spatial Games of Fake News - Supplementary Information

Matthew I Jones, Scott D Pauls, Feng Fu

This supplementary information contains an additional exploration of the fake news spatial game described in the main paper, as well as the derivation of the invasion probabilities in the limit of weak selection.

1 Echo Chamber Longevity and the Pseudo-steady State

In this section, we expand our investigation into the role sanctioners play in containing the spread of fake news. Specifically, we explore how the density of static sanctioners impacts the formation of echo chambers and the dominant strategy that controls over half of the viable population. As illustrated in Fig S1a and S1b for the square lattice, the dominating strategy varies with the sanctioner density.

While our main paper focused on a critical value of p_C at which real news becomes favored over fake news, we also consider another important point: eradicating fake news, instead of just containing it, by recruiting enough sanctioners to eliminate fake news everywhere. This is a more difficult task, as it requires enough sanctioners to break up echo chambers wherever they may form. As a result, there are four distinct regions of behavior: fake news (B) fixating and real news (A) going extinct, fake news having the advantage in the population with small real news echo chambers, real news having the advantage with small fake news echo chambers, and real news fixating with fake news going extinct. This sequence of behaviors and their probabilities are shown in Fig S1c.

We can see the formation of echo chambers for a wide range of sanctioner densities, approximately 0.15 to 0.5 in the case of the square lattice with selection strength $\beta = 0.5$. We call behavior in this region the pseudo-steady state because these echo chambers are highly resistant to invasion and thus can persist for millions of time steps. However, it is not a true steady state because with an infinite amount of time, eventually the echo chambers will break down and one strategy will go extinct.

We can see the resilience of these echo chambers by looking at the number of real news sharers as a function of time. Figure S2a shows the prevalence

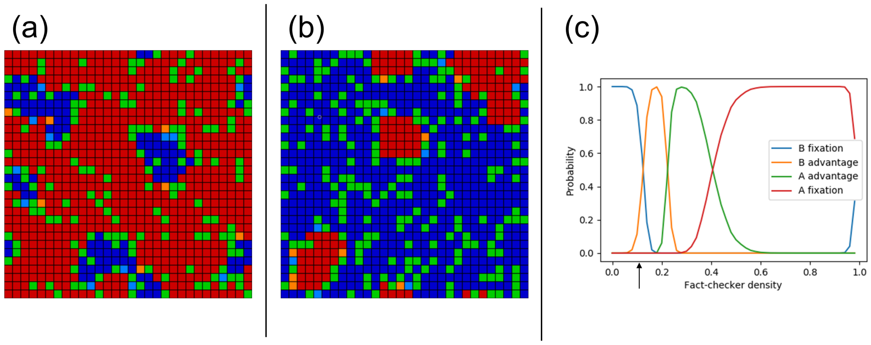

Figure S1: Panels a and b show echo chambers consisting of individuals who share either real news (blue) or fake news (red), separated from the rest of the population by sanctioners (green). Lightly-colored individuals are those that have changed strategy in the last time step. The plot in (c) uses simulations to show how the long-term behavior changes as the sanctioner density varies, with the arrow indicating the critical sanctioner density at which real news has an advantage in a well-mixed population ($p_C = 1/11$). As the number of sanctioners increases, the population moves towards more real news and less false news stories being shared.

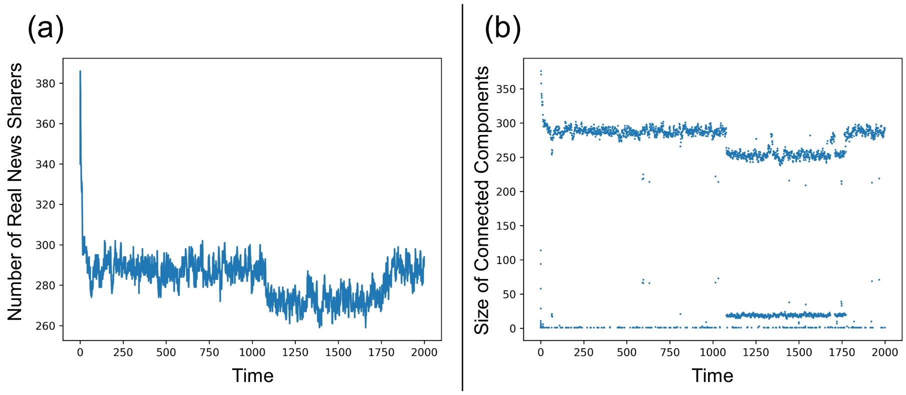

Figure S2: The characteristic evolution of a 900 individual population with $p_C = 0.2$ over the course of 2000 time steps. In (a), we can see that after a short chaotic period, the system reaches a pseudo-steady state and the number of true news sharers is fairly constant except for short bursts of disruption when clusters of individuals all shift strategy together. In (b), we get a more detailed look at what happened in the same system by looking at the size of individual connected components. Around $t = 1100$, the single large component of real news sharers splits into two separate components. Then at about $t = 1800$, the two components are joined together as a small cluster between them changes back to sharing real news.

of real news in a single representative simulation. The number of cooperators drops swiftly at first before stabilizing at around 290 cooperators. There are small shifts at $t \approx 1100$ and $t \approx 1800$, but otherwise the population is unchanging except for minor perturbations on the border of echo chambers. Fig S2b gives more detail, showing the size of each path-connected component of real news sharers. Notably, changes in cooperator population size correspond to the formation and fusion of two smaller components (one with approximately 250 individuals and the other with approximately 20) into the large 290 individual cluster.

On the square lattice, the formation of echo chambers and the pseudo-steady state seems to occur across a wide range of sanctioner densities. As shown in the main paper, we also observe echo chamber formation on small-world networks and the twitter network. However, this is not a uniform property of all networks. Preliminary results show that the formation of echo chambers and the critical p_C value are dependent on network topology; lattices and small-worlds are fertile ground for echo chambers, but Erdös-Renyí random graphs and scale-free networks are not. This leads us to hypothesize that a relatively high clustering coefficient is essential for the formation of echo chambers. This intuitively makes sense, as echo chambers are dependent on the feedback loops possible in cliquish, highly connected communities.

2 Sanctioner Inaccuracy

In reality, sanctioning is subject to human errors. Some fake news occasionally goes unnoticed and endorsed, and some real news is temporally labelled to be fake by well-meaning sanctioners. Moreover, when relying on citizens instead of professional journalists for peer policing, the accuracy of fact-checking will inevitably go down as laymen are less prepared to accurately assess fake news. Suppose that sanctioners have an accuracy in their policing of $\lambda \in [0, 1]$. With probability λ, they correctly assess a post's accuracy and reward benefit α to true news spreaders and penalty γ to fake news spreaders. With probability $1-\lambda$, an error occurs, leading to the opposite payoff assignments. Using the same method, we use to calculate the analytic fixation probabilities, we will quantify the precision threshold required for sanctioners to ensure fair and transparent policing of wrongdoers while in favor of real news spreaders. For the exact expressions, see the end of the section on analytic derivations below. Figure S3 shows the relationship between invasion probabilities on the $p_C - \lambda$ plane when using the following payoff matrix:

$$\begin{array}{c} \\ A \\ B \\ C \end{array} \begin{array}{c} \begin{array}{ccc} A & B & C \end{array} \\ \begin{pmatrix} 1 & 0 & \lambda - 4(1-\lambda) \\ 0 & 2 & -4\lambda + (1-\lambda) \\ 0 & 0 & 0 \end{pmatrix} \end{array} \tag{1}$$

In Fig S3a, we see that when $\lambda < 0.5$, selection always favors fake news. This is unsurprising, as it means that the supposed fact-checkers are actually giving

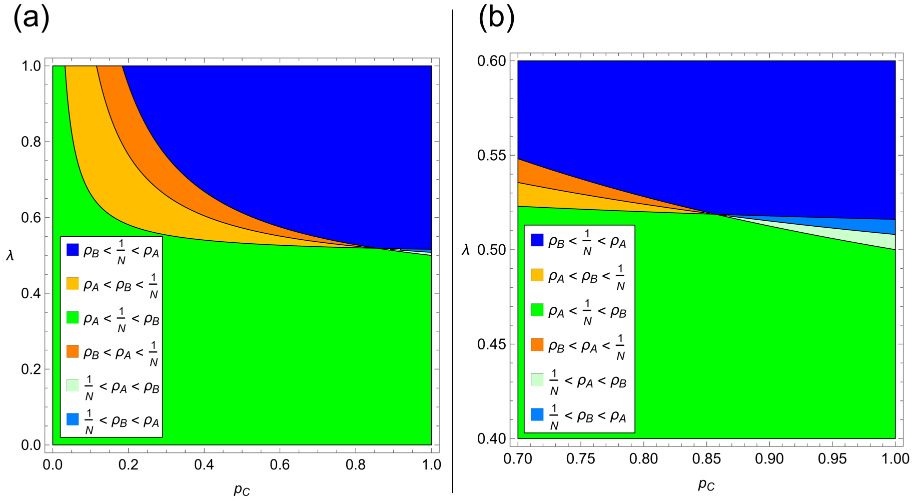

Figure S3: The results of varying the accuracy of sanctioners. In (a), we see the where in the $p_C - \lambda$ plane selection favors true news (blue), false news (green), or neither (orange). However, when the density of sanctioners is very high and sanctioners are not very accurate, selection can actually favor invasion by true or false news, as shown in (b). This is surprising because this is a coordination game and it is rare for selection to favor invasion by both strategies. However, this combination of parameter values is highly unrealistic and would never occur in real life.

more benefit to fake news spreaders than real news spreaders. However, there is a clear buffer in which sanctioners can be accurate only about 80% of the time without necessitating a drastic increase in the critical sanctioner density for selection to favor real news.

Fig S3b shows an interesting phenomenon. When sanctioner accuracy is very close to 1/2 and the number of sanctioners is extremely high, selection actually favors invasion by both real *and* fake news. This is surprising because this real vs fake news game is a coordination game which tends to oppose invading mutants. While this set of parameters is unrealistic and would never appear in any real population, it still demonstrates an interesting property of the dynamics of coordination games in the presence of zealots or extreme environmental conditions.

3 Robustness of Model Parameters and Initial Conditions

The primary model presented in this paper makes some assumptions about the system. In this section, we test the robustness of our results to perturbations of these parameters and show that the qualitative results remain for almost all parameter values. We focus on three parameters: the $B - B$ payoff (2 in the main paper), the $B - C$ punishment (-4 in the main paper), and the initial ratio of A to B players ($\frac{1}{2}$ in the main paper. For all three parameters, we use simulations to test how the critical sanctioner density changes on the grid and the small-world networks while holding the other parameters constant. We also show the impact in the well-mixed model under replicator dynamics.

3.1 Initial Distribution of Sharers of Real versus Fake News

One property of our model is that it is initialized with an equal number of A and B players. As we mentioned, this is unrealistic, with fake news spreaders making up a very small fraction of the population. We vary this initial distribution from entirely real news ($\frac{p_A}{p_A+p_B} = \frac{p_A}{1-p_C} = 1$) to entirely fake news ($\frac{p_A}{1-p_C} = 0$).

To find a closed-form solution for the critical sanctioner density in the well-mixed case, we once again begin with ensuring that the fitness of A and B players is the same.

For interpretability, the independent variable we are interested in is not p_A, but $\frac{p_A}{1-p_C}$, which we denote as x for readability.

$$f_A = 1(1 - p_C)x + 1(p_C) \tag{2}$$

$$f_B = 2(1 - p_C)(1 - x) - 4(p_C) \tag{3}$$

Now we set these equal and solve for p_C.

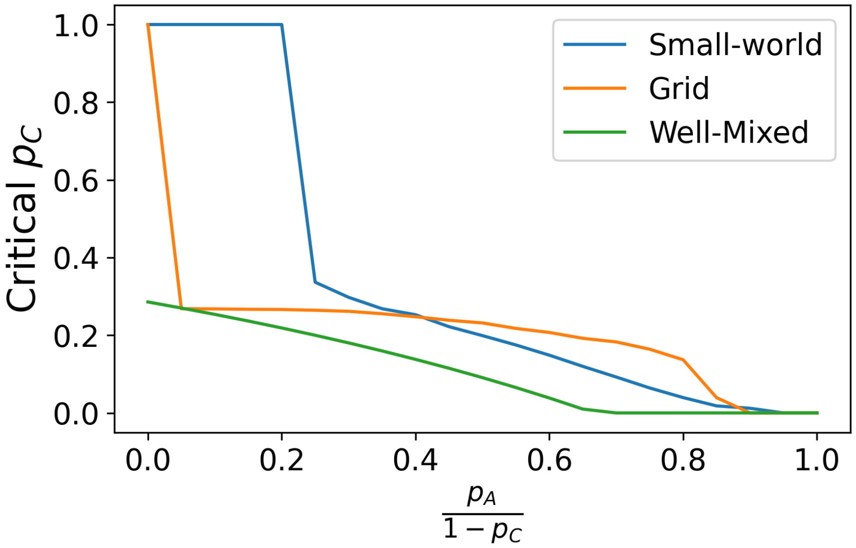

Figure S4: The effect of changing the initial ratio of A and B players on the critical sanctioner density. Notice that the results are quite different in the small-world and lattice networks, and different networks are better or worse for fake news depending on the initial distribution. The positive portion of the green curve shows the analytic results for the well-mixed case in Equation (5). Simulation estimates were done with 200 iterations at 20 evenly-spaced values of $\frac{p_A}{1-p_C}$ and adjusting p_C by step size 0.025.

$$1(1-p_C)x + 1(p_C) = 2(1-p_C)(1-x) - 4(p_C) \tag{4}$$

$$p_C = \frac{x - 2(1-x)}{-5 + x - 2(1-x)} = \frac{3x-2}{3x-7} \tag{5}$$

Figure S4 shows the plot of Equation (5), at least until it becomes negative, indicating that the critical sanctioner density is zero.

3.2 The $B - B$ Payoff Value

Another assumption of our model is the inherent advantage the false B narrative has in the payoff matrix, where $B - B$ interactions provide twice as much value as $A - A$ interactions. There are many reasons this could be true, but this is still a contested issue. Thus, we also test our results for values ranging from 1 to 3.

When the $B - B$ payoff is the same as the $A - A$ payoff, neither group has any advantage so the critical value of p_C is zero for both the well-mixed and networked models. However, as shown in Figure S5, once B has any advantage over A, we see the network structure of both grids and lattices giving B a significant protection compared to the well-mixed case.

Using the same techniques as above, it is straightforward to see that in the well-mixed case, if d is the $B-B$ payoff (see Equation (8)), the critical sanctioner density is given by

$$p_C = \frac{d-1}{9+d} \tag{6}$$

3.3 The $B - C$ Punishment Value

Finally, we also consider the punishment that sanctioners inflict on fake news sharers. In the main model, we assume this is twice the benefit that fake news sharers provide to each other, but this is a very difficult parameter to estimate in a rigorous way. Therefore, we also consider what happens as we adjust this parameter from a very strong punishment (-8) to no punishment, where the only help sanctioners provide A players is an unwavering source of support. Surprisingly, networked populations need about 10-20% more sanctioners than the well-mixed case to contain fake news, regardless of the punishment factor.

If we label the punishment term γ like Equation (8), the well-mixed case has a closed form solution

$$p_C = \frac{1}{3 - 2\gamma} \tag{7}$$

which is plotted in Figure S6.

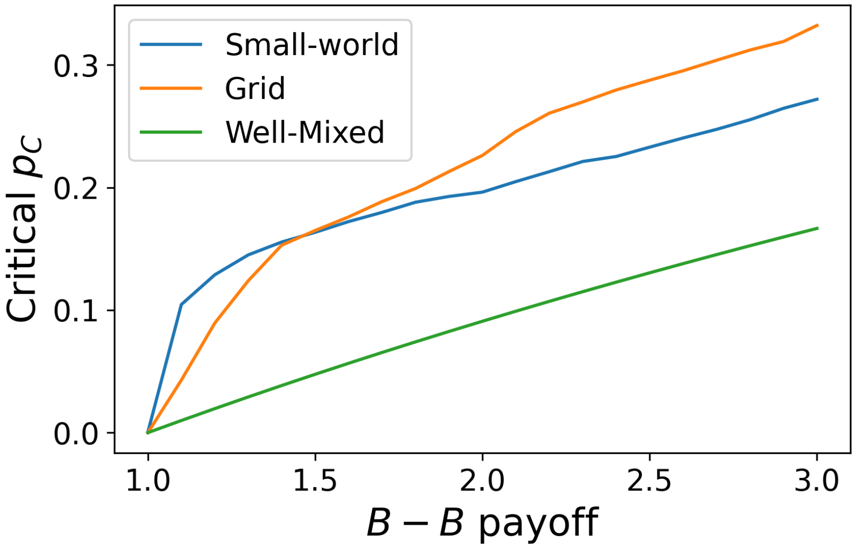

Figure S5: The effect of changing the $B - B$ payoff on the critical sanctioner density. For small advantages, fake news is stronger in small-world networks, but when the advantage is large, the grid network gives fake news a stronger effect. The green curve shows the analytic results for the well-mixed case in Equation (6). Simulation estimates were done with 200 iterations at 20 evenly-spaced values of $\frac{p_A}{1-p_C}$ and adjusting p_C by step size 0.025.

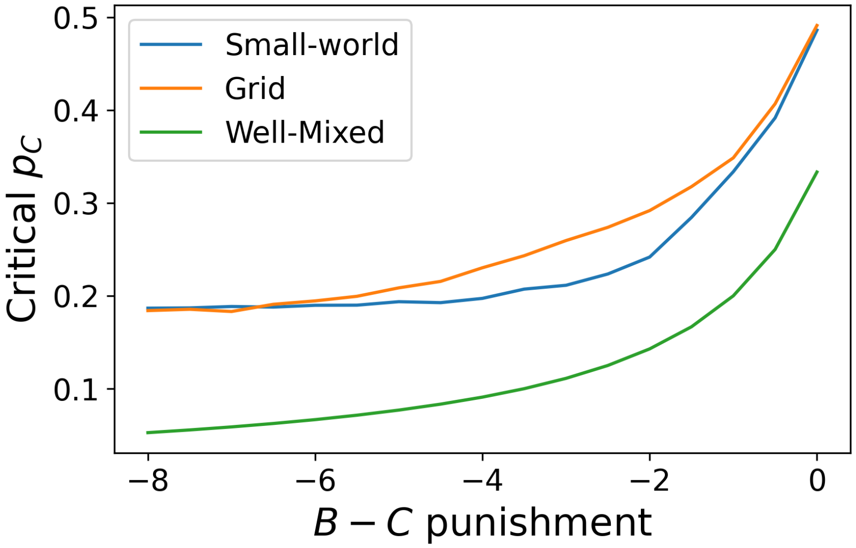

Figure S6: The effect of changing the $B-C$ punishment on the critical sanctioner density. The curves for the two networks grow in tandem with the well-mixed case, so we conclude that increasing the punishment term does very little to address the problem of localized sanctioning. The green curve shows the analytic results for the well-mixed case given in Equation (7). Simulation estimates were done with 200 iterations at 16 evenly-spaced values for the punishment parameter and adjusting p_C by step size 0.025.

4 Derivation of Analytic Results

In this section, we derive the invasion probabilities of single cooperators and defectors in the limit of weak selection. We begin by introducing the necessary notation. We have N individuals on a network, each with k neighbors, and they play a game with a general payoff matrix

$$\begin{array}{c} \\ A \\ B \\ C \end{array} \begin{array}{c} \begin{matrix} A & B & C \end{matrix} \\ \begin{pmatrix} a & b & \alpha \\ c & d & \gamma \\ 0 & 0 & 0 \end{pmatrix} \end{array} \tag{8}$$

p_A, p_B, and p_C are the proportions of A, B, and C players. Similarly, $p_{S_1 S_2}$ is the proportion of edges leading from an individual playing S_1 to an individual playing S_2, where S_1 and S_2 can be A, B, or C. We will also be interested in the conditional probability of finding an individual playing S_2 by following a random edge that starts at an individual playing S_1, which will be denoted $q_{S_2|S_1}$. By basic probability, $q_{S_2|S_1} = \frac{p_{S_1 S_2}}{p_{S_1}}$.

For an individual playing S_i, π_{S_i} is the total payoff, or the sum of the payoffs from each interaction with a neighbor. The payoff of any A or B individual is dependent on the neighbors' strategies, but we are interested in the expected payoff which only depends on the quantities already listed. With selection strength β, $f_{S_i} = e^{\beta \pi_{S_i}}$ is the fitness of an individual playing S_i.

We have two normalization conditions that ensure that all our probabilities sum to 1:

$$p_A + p_B + p_C = 1 \tag{9}$$

$$p_{AA} + p_{AB} + p_{AC} + p_{BA} + p_{BB} + p_{BC} + p_{CA} + p_{CB} + p_{CC} = 1 \tag{10}$$

Additionally, there are three symmetry conditions. These need not be true in general, but because the network we are using is undirected, an edge from S_1 to S_2 is also an edge from S_2 to S_1. Therefore:

$$p_{AB} = p_{BA} \tag{11}$$

$$p_{AC} = p_{CA} \tag{12}$$

$$p_{BC} = p_{CB} \tag{13}$$

Finally, we have three consistency conditions:

$$p_A = p_{AA} + p_{AB} + p_{AC} \tag{14}$$

$$p_B = p_{BA} + p_{BB} + p_{BC} \tag{15}$$

$$p_C = p_{CA} + p_{CB} + p_{CC} \tag{16}$$

With all these conditions, we can simplify the system until there are only five independent variables: p_A, p_B, p_{AA}, p_{BB}, p_{CC}. The other four variables can be solved in terms of these five:

$$p_C = 1 - p_A - p_B \tag{17}$$

$$p_{AB} = p_{BA} = 1/2\Big[(p_A - p_{AA}) + (p_B - p_{BB}) - (p_C - p_{CC})\Big] \quad (18)$$

$$p_{AC} = p_{CA} = 1/2\Big[(p_C - p_{CC}) - (p_B - p_{BB}) + (p_A - p_{AA})\Big] \quad (19)$$

$$p_{BC} = p_{CB} = 1/2\Big[(p_B - p_{BB}) + (p_C - p_{CC}) - (p_A - p_{AA})\Big] \quad (20)$$

Now we are ready to derive differential equations for the systems evolution in time.

4.1 Pair Approximation

The game between real and fake news is a coordination game, and because of this, individuals will tend to form clusters of like-minded individuals, as observed in simulations. However, because of this, the probabilities along two successive edges are not independent. That is to say, if $p_{S_1S_2S_3}$ is the probability of starting at an S_1 player, following a random edge to an S_2 player, and then following another random edge to an S_3 player, we do **not** get that

$$p_{S_1S_2S_3} = \frac{p_{S_1S_2}p_{S_2S_3}}{p_{S_2}} \quad (21)$$

However, this makes studying the system untenable. Pair approximation alleviates this problem by making the simplifying assumptions that edges are independent and therefore Equation (21) holds.

In the death-birth process, an individual is chosen to "die" and a neighbor is chosen to replicate and take the deceased individuals place. However, if the two individuals are playing the same strategy, nothing in the population will have changed. The only way the system changes is if an A individual takes the place of a B individual or vice versa, so we focus on the frequency of these two events to study the system.

We use the modified update step where only one individual is replaced per time step. This slows down the system's evolution by a factor of $\frac{1}{N}$, but it has very little effect on the behavior of the system, and it makes the system much easier to approach analytically. With a discrete time step $\Delta t = \frac{1}{N}$ so that one individual is replaced per time step, the differential equations for p_A and p_{AA} are:

$$\dot{p_A} = \frac{1}{N}\frac{E(\Delta n_A)}{\Delta t} = E(\Delta n_A) \quad (22)$$

$$\dot{p_{AA}} = \frac{2}{kN}\frac{E(\Delta n_{AA})}{\Delta t} = \frac{2}{k}E(\Delta n_{AA}) \quad (23)$$

We first focus on computing $E(\Delta n_A)$. Because only one individual updates at a time, $E(\Delta n_A) = P(\Delta n_A = 1) - P(\Delta n_A = -1)$. n_A increases by one when a B player is replaced by an A player, and n_A decreases by one when an A player is replaced by a B player. We now derive the probability of an A player replacing a B player. The probability of B invading A follows by symmetry.

The B player that is being replaced has k neighbors, each of which can be an A, B, or C player. Specifically, the focal B player has k_B^A A neighbors, k_B^B B neighbors, and k_B^C C neighbors with probability

$$\frac{k!}{k_B^A!k_B^B!k_B^C!} q_{A|B}^{k_B^A} q_{B|B}^{k_B^B} q_{C|B}^{k_B^C} \tag{24}$$

and there is always the restriction that $k_B^A + k_B^B + k_B^C = k$.

Each of these neighbors has $k-1$ neighbors (not including the focal B player) that are also multinomially distributed. An A-playing neighbor will have ${k'}_A^A$ A neighbors, ${k'}_A^B$ B neighbors, and ${k'}_A^C$ C neighbors with probability

$$\frac{(k-1)!}{{k'}_A^A!{k'}_A^B!{k'}_A^C!} q_{A|A}^{{k'}_A^A} q_{B|A}^{{k'}_A^B} q_{C|A}^{{k'}_A^C} \tag{25}$$

Here we used pair approximation, because we ignore the higher-order terms that might arise knowing that the A player already has a B neighbor.

Likewise, the B and C players neighboring the focal B player have neighbors whose strategies are multinomially distributed. To determine the strategy the focal B player will choose to imitate, we need to know the payoffs of all of the neighbors.

An A neighbor of the focal B player who has ${k'}_A^A$ A neighbors, ${k'}_A^B$ B neighbors (not including the focal B player), and ${k'}_A^C$ C neighbors has payoff

$$\pi_A = {k'}_A^A a + ({k'}_A^B + 1)b + {k'}_A^C \alpha \tag{26}$$

and fitness

$$f_A({k'}_A^A, {k'}_A^B, {k'}_A^C) = e^{\beta \pi_A} \tag{27}$$

The same quantities for the B and C neighbors work the same way.

$$\pi_B = {k'}_B^A c + ({k'}_B^B + 1)d + {k'}_B^C \gamma \tag{28}$$

$$f_B({k'}_B^A, {k'}_B^B, {k'}_B^C) = e^{\beta \pi_B} \tag{29}$$

$$\pi_C = {k'}_C^A 0 + ({k'}_C^B + 1)0 + {k'}_C^C 0 = 0 \tag{30}$$

$$f_C({k'}_C^A, {k'}_C^B, {k'}_C^C) = e^{\beta \pi_C} = 1 \tag{31}$$

We are interested in the focal B player being replaced by an A player. Because individuals choose who to copy proportional to fitness, the probability of the B player selecting one of its A neighbors is

$$\frac{k_B^A f_A}{k_B^A f_A + k_B^B f_B + k_B^C f_C} \tag{32}$$

All that remains is to sum over all possible configurations of the B player's neighbors and their neighbors and multiply by p_B (the probability that a B

player is selected to update) to get the final probability W_{AB} that a B player is replaced by an A player:

$$
\begin{aligned}
W_{AB} = p_B \cdot & \sum_{k_B^A+k_B^B+k_B^C=k} \frac{k!}{k_B^A!k_B^B!k_B^C!} q_{A|B}^{k_B^A} q_{B|B}^{k_B^B} q_{C|B}^{k_B^C} \\
\cdot & \sum_{k'^A_A+k'^B_A+k'^C_A=k-1} \frac{(k-1)!}{k'^A_A!k'^B_A!k'^C_A!} q_{A|A}^{k'^A_A} q_{B|A}^{k'^B_A} q_{C|A}^{k'^C_A} \\
\cdot & \sum_{k'^A_B+k'^B_B+k'^C_B=k-1} \frac{(k-1)!}{k'^A_B!k'^B_B!k'^C_B!} q_{A|B}^{k'^A_B} q_{B|B}^{k'^B_B} q_{C|B}^{k'^C_B} \\
\cdot & \sum_{k'^A_C+k'^B_C+k'^C_C=k-1} \frac{(k-1)!}{k'^A_C!k'^B_C!k'^C_C!} q_{A|C}^{k'^A_C} q_{B|C}^{k'^B_C} q_{C|C}^{k'^C_C} \\
\cdot & \frac{k_B^A f_A(k'^A_A, k'^B_A+1, k'^C_A)}{k_B^A f_A(k'^A_A, k'^B_A+1, k'^C_A) + k_B^B f_B(k'^A_B, k'^B_B+1, k'^C_B) + k_B^C f_C(k'^A_C, k'^B_C+1, k'^C_C)}
\end{aligned} \tag{33}
$$

(Though it is difficult to typeset within the margins, note that this is a nested sum and not the product of four separate sums.) Likewise, W_{BA}, the probability of B invading A, is

$$
\begin{aligned}
W_{BA} = p_A \cdot & \sum_{k_A^A+k_A^B+k_A^C=k} \frac{k!}{k_A^A!k_A^B!k_A^C!} q_{A|A}^{k_A^A} q_{B|A}^{k_A^B} q_{C|A}^{k_A^C} \\
\cdot & \sum_{k'^A_A+k'^B_A+k'^C_A=k-1} \frac{(k-1)!}{k'^A_A!k'^B_A!k'^C_A!} q_{A|A}^{k'^A_A} q_{B|A}^{k'^B_A} q_{C|A}^{k'^C_A} \\
\cdot & \sum_{k'^A_B+k'^B_B+k'^C_B=k-1} \frac{(k-1)!}{k'^A_B!k'^B_B!k'^C_B!} q_{A|B}^{k'^A_B} q_{B|B}^{k'^B_B} q_{C|B}^{k'^C_B} \\
\cdot & \sum_{k'^A_C+k'^B_C+k'^C_C=k-1} \frac{(k-1)!}{k'^A_C!k'^B_C!k'^C_C!} q_{A|C}^{k'^A_C} q_{B|C}^{k'^B_C} q_{C|C}^{k'^C_C} \\
\cdot & \frac{k_A^B f_B(k'^A_B+1, k'^B_B, k'^C_B)}{k_A^A f_A(k'^A_A+1, k'^B_A, k'^C_A) + k_A^B f_B(k'^A_B+1, k'^B_B, k'^C_B) + k_A^C f_C(k'^A_C+1, k'^B_C, k'^C_C)}
\end{aligned} \tag{34}
$$

Furthermore, when B is invaded by A it increases the number of $A-A$ pairs by k_B^A, so we can define ϕ_{AB}^A to be the expected value for the change in $A-A$ edges due to a B player being invaded by an A player. (The subscript describes the direction of invasion and the superscript determines which pair it corresponds to, so ϕ_{AB}^A means an A player is replacing a B player, and this term tells us about the change in $A-A$ pairs.) Like in (33), we have

$$
\begin{aligned}
\phi_{AB}^{A} = p_B \cdot & \sum_{k_B^A+k_B^B+k_B^C=k} k_B^A \frac{k!}{k_B^A! k_B^B! k_B^C!} q_{A|B}^{k_B^A} q_{B|B}^{k_B^B} q_{C|B}^{k_B^C} \\
\cdot & \sum_{k'^A_A+k'^B_A+k'^C_A=k-1} \frac{(k-1)!}{k'^A_A! k'^B_A! k'^C_A!} q_{A|A}^{k'^A_A} q_{B|A}^{k'^B_A} q_{C|A}^{k'^C_A} \\
\cdot & \sum_{k'^A_B+k'^B_B+k'^C_B=k-1} \frac{(k-1)!}{k'^A_B! k'^B_B! k'^C_B!} q_{A|B}^{k'^A_B} q_{B|B}^{k'^B_B} q_{C|B}^{k'^C_B} \\
\cdot & \sum_{k'^A_C+k'^B_C+k'^C_C=k-1} \frac{(k-1)!}{k'^A_C! k'^B_C! k'^C_C!} q_{A|C}^{k'^A_C} q_{B|C}^{k'^B_C} q_{C|C}^{k'^C_C} \\
\cdot & \frac{k_B^A f_A(k'^A_A, k'^B_A+1, k'^C_A)}{\begin{aligned} k_B^A f_A(k'^A_A, k'^B_A+1, k'^C_A) + k_B^B f_B(k'^A_B, k'^B_B+1, k'^C_B) \\ + k_B^C f_C(k'^A_C, k'^B_C+1, k'^C_C) \end{aligned}}
\end{aligned}
\tag{35}
$$

Note that (35) only differs from (33) in a single k_B^A term in the first line, which is there because we are interested in the expected value of the change in $A-A$ edges, and there are k_B^A new $A-A$ edges being formed. Similarly, we can write down:

$$
\begin{aligned}
\phi_{BA}^{A} = p_A \cdot & \sum_{k_A^A+k_A^B+k_A^C=k} k_A^A \frac{k!}{k_A^A! k_A^B! k_A^C!} q_{A|A}^{k_A^A} q_{B|A}^{k_A^B} q_{C|A}^{k_A^C} \\
\cdot & \sum_{k'^A_A+k'^B_A+k'^C_A=k-1} \frac{(k-1)!}{k'^A_A! k'^B_A! k'^C_A!} q_{A|A}^{k'^A_A} q_{B|A}^{k'^B_A} q_{C|A}^{k'^C_A} \\
\cdot & \sum_{k'^A_B+k'^B_B+k'^C_B=k-1} \frac{(k-1)!}{k'^A_B! k'^B_B! k'^C_B!} q_{A|B}^{k'^A_B} q_{B|B}^{k'^B_B} q_{C|B}^{k'^C_B} \\
\cdot & \sum_{k'^A_C+k'^B_C+k'^C_C=k-1} \frac{(k-1)!}{k'^A_C! k'^B_C! k'^C_C!} q_{A|C}^{k'^A_C} q_{B|C}^{k'^B_C} q_{C|C}^{k'^C_C} \\
\cdot & \frac{k_A^B f_B(k'^A_B+1, k'^B_B, k'^C_B)}{\begin{aligned} k_A^A f_A(k'^A_A+1, k'^B_A, k'^C_A) + k_A^B f_B(k'^A_B+1, k'^B_B, k'^C_B) \\ + k_A^C f_C(k'^A_C+1, k'^B_C, k'^C_C) \end{aligned}}
\end{aligned}
\tag{36}
$$

$$
\begin{aligned}
\phi^B_{AB} = p_B \cdot & \sum_{k^A_B + k^B_B + k^C_B = k} k^B_B \frac{k!}{k^A_B! k^B_B! k^C_B!} q^{k^A_B}_{A|B} q^{k^B_B}_{B|B} q^{k^C_B}_{C|B} \\
\cdot & \sum_{k'^A_A + k'^B_A + k'^C_A = k-1} \frac{(k-1)!}{k'^A_A! k'^B_A! k'^C_A!} q^{k'^A_A}_{A|A} q^{k'^B_A}_{B|A} q^{k'^C_A}_{C|A} \\
\cdot & \sum_{k'^A_B + k'^B_B + k'^C_B = k-1} \frac{(k-1)!}{k'^A_B! k'^B_B! k'^C_B!} q^{k'^A_B}_{A|B} q^{k'^B_B}_{B|B} q^{k'^C_B}_{C|B} \\
\cdot & \sum_{k'^A_C + k'^B_C + k'^C_C = k-1} \frac{(k-1)!}{k'^A_C! k'^B_C! k'^C_C!} q^{k'^A_C}_{A|C} q^{k'^B_C}_{B|C} q^{k'^C_C}_{C|C} \\
\cdot & \frac{k^A_B f_A(k'^A_A, k'^B_A + 1, k'^C_A)}{k^A_B f_A(k'^A_A, k'^B_A + 1, k'^C_A) + k^B_B f_B(k'^A_B, k'^B_B + 1, k'^C_B) + k^C_B f_C(k'^A_C, k'^B_C + 1, k'^C_C)}
\end{aligned} \tag{37}
$$

$$
\begin{aligned}
\phi^B_{BA} = p_A \cdot & \sum_{k^A_A + k^B_A + k^C_A = k} k^B_A \frac{k!}{k^A_A! k^B_A! k^C_A!} q^{k^A_A}_{A|A} q^{k^B_A}_{B|A} q^{k^C_A}_{C|A} \\
\cdot & \sum_{k'^A_A + k'^B_A + k'^C_A = k-1} \frac{(k-1)!}{k'^A_A! k'^B_A! k'^C_A!} q^{k'^A_A}_{A|A} q^{k'^B_A}_{B|A} q^{k'^C_A}_{C|A} \\
\cdot & \sum_{k'^A_B + k'^B_B + k'^C_B = k-1} \frac{(k-1)!}{k'^A_B! k'^B_B! k'^C_B!} q^{k'^A_B}_{A|B} q^{k'^B_B}_{B|B} q^{k'^C_B}_{C|B} \\
\cdot & \sum_{k'^A_C + k'^B_C + k'^C_C = k-1} \frac{(k-1)!}{k'^A_C! k'^B_C! k'^C_C!} q^{k'^A_C}_{A|C} q^{k'^B_C}_{B|C} q^{k'^C_C}_{C|C} \\
\cdot & \frac{k^B_A f_B(k'^A_B + 1, k'^B_B, k'^C_B)}{k^A_A f_A(k'^A_A + 1, k'^B_A, k'^C_A) + k^B_A f_B(k'^A_B + 1, k'^B_B, k'^C_B) + k^C_A f_C(k'^A_C + 1, k'^B_C, k'^C_C)}
\end{aligned} \tag{38}
$$

Once we have these quantities (Equations (33) - (38)), we have expressions for all of our independent variables.

$$\dot{p_{CC}} = 0 \tag{39}$$

$$\dot{p_A} = -\dot{p_B} = W_{AB} - W_{BA} \tag{40}$$

$$\dot{p_{AA}} = \frac{2}{k}(\phi^A_{AB} - \phi^A_{BA}) \tag{41}$$

$$\dot{p_{BB}} = \frac{2}{k}(\phi^B_{BA} - \phi^B_{AB}) \tag{42}$$

4.2 Weak Selection

Even with the substantial simplification from pair approximation, the previous results are too complicated and unwieldy to be useful by themselves. Because of compounding sums, directly calculating the derivatives requires adding millions of terms if $k = 8$. Furthermore, the pair approximation means that we lose the information critical to clustering, and therefore the analytic results here will fail to capture the pseudo-steady states that we observe when β is much larger than zero.

We can sidestep both these issues by working in the limit of weak selection. In weak selection, the success or failure of an individual in the fake news game is only one small factor in the individual's success, and fitnesses are much more uniform across the population. When β is close to zero, we can throw out higher order terms which simplifies the expression, and when β is close to zero, the pseudo-steady states cannot exist anyways because the system behaves approximately like neutral drift. Taking the Taylor expansion of the exponential in equations (27) and (29) with respect to β and only keeping the low order terms, what is left is mathematically tractable. We have expressions for each of $W_{AB}, W_{BA}, \phi^A_{AB}, \phi^A_{BA}, \phi^B_{BA}$, and ϕ^B_{AB}. We manipulate each separately and bring them back together at the end.

4.3 W_{AB} and W_{BA}:

Equation (33) gives us an expression for W_{AB}. The individuals playing C have constant fitness, $f_C = 1$, and no other terms in the last line of (33) depend on the neighbors of C players, so we can pull it all through the final sum which collapses to 1 because it is the sum of the probabilities of all possible configurations of neighbors, which must be 1. Therefore,

$$
\begin{aligned}
W_{AB} = p_B \cdot & \sum_{k_B^A + k_B^B + k_B^C = k} \frac{k!}{k_B^A! k_B^B! k_B^C!} q_{A|B}^{k_B^A} q_{B|B}^{k_B^B} q_{C|B}^{k_B^C} \\
\cdot & \sum_{k_A^{A'} + k_A^{B'} + k_A^{C'} = k-1} \frac{(k-1)!}{k_A^{A'}! k_A^{B'}! k_A^{C'}!} q_{A|A}^{k_A^{A'}} q_{B|A}^{k_A^{B'}} q_{C|A}^{k_A^{C'}} \\
\cdot & \sum_{k_B^{A'} + k_B^{B'} + k_B^{C'} = k-1} \frac{(k-1)!}{k_B^{A'}! k_B^{B'}! k_B^{C'}!} q_{A|B}^{k_B^{A'}} q_{B|B}^{k_B^{B'}} q_{C|B}^{k_B^{C'}} \\
\cdot & \frac{k_B^A f_A(k_A^{A'}, k_A^{B'} + 1, k_A^{C'})}{k_B^A f_A(k_A^{A'}, k_A^{B'} + 1, k_A^{C'}) + k_B^B f_B(k_B^{A'}, k_B^{B'} + 1, k_B^{C'}) + k_B^C}
\end{aligned}
\tag{43}
$$

Then, using the Taylor expansion for the exponentials in f_A and f_B but only keeping the low order terms of β, we have

$$\begin{aligned}
&\frac{k_B^A f_A(k_A^{A'}, k_A^{B'}+1, k_A^{C'})}{k_B^A f_A(k_A^{A'}, k_A^{B'}+1, k_A^{C'}) + k_B^B f_B(k_B^{A'}, k_B^{B'}+1, k_B^{C'}) + k_B^C} \\
&\approx \frac{k_B^A\big(1+\beta(ak_A^{A'} + b(k_A^{B'}+1) + \alpha k_A^{C'})\big)}{\begin{array}{l} k_B^A\big(1+\beta(ak_A^{A'} + b(k_A^{B'}+1) + \alpha k_A^{C'})\big) \\ \quad + k_B^B\big(1+\beta(ck_B^{A'} + d(k_B^{B'}+1) + \gamma k_B^{C'})\big) + k_B^C \end{array}} \\
&= \frac{k_B^A(1+\beta u_1)}{k + \beta(k_B^A u_1 + k_B^B u_2)} \\
&\approx k_B^A(1+\beta u_1)[\frac{1}{k} - \frac{k_B^A u_1 + k_B^B u_2}{k^2}\beta] \\
&\approx \frac{k_B^A}{k} + \beta[\frac{k_B^A u_1}{k} - k_B^A \frac{k_B^A u_1 + k_B^B u_2}{k^2}]
\end{aligned} \tag{44}$$

where $u_1 = ak_A^{A'} + b(k_A^{B'}+1) + \alpha k_A^{C'}$ and $u_2 = ck_B^{A'} + d(k_B^{B'}+1) + \gamma k_B^{C'}$. By carefully pulling terms through the sums, we have the following identities:

$$\begin{aligned}
&\sum_{k_A^{A'}+k_A^{B'}+k_A^{C'}=k-1} \frac{(k-1)!}{k_A^{A'}!k_A^{B'}!k_A^{C'}!} q_{A|A}^{k_A^{A'}} q_{B|A}^{k_A^{B'}} q_{C|A}^{k_A^{C'}} u_1 \\
&\qquad = a(k-1)q_{A|A} + b\big((k-1)q_{B|A}+1\big) + \alpha(k-1)q_{C|A} \\
&\qquad = E_A + b
\end{aligned} \tag{45}$$

$$\begin{aligned}
&\sum_{k_B^{A'}+k_B^{B'}+k_B^{C'}=k-1} \frac{(k-1)!}{k_B^{A'}!k_B^{B'}!k_B^{C'}!} q_{A|B}^{k_B^{A'}} q_{B|B}^{k_B^{B'}} q_{C|B}^{k_B^{C'}} u_2 \\
&\qquad = c(k-1)q_{A|B} + d\big((k-1)q_{B|B}+1\big) + \gamma(k-1)q_{C|B} \\
&\qquad = E_B + d
\end{aligned} \tag{46}$$

Notice that E_A and E_B are the expected payoffs for A and B players from $k-1$ neighbors. Using these identities on our equation for W_{AB}, we get that

$$\begin{aligned}
W_{AB} = p_B \cdot &\sum_{k_B^A+k_B^B+k_B^C=k} \frac{k!}{k_B^A!k_B^B!k_B^C!} q_{A|B}^{k_B^A} q_{B|B}^{k_B^B} q_{C|B}^{k_B^C} \\
&\cdot \left[\frac{k_B^A}{k} - \beta\frac{k_B^B k_B^A}{k^2}(E_B+d) + \beta\frac{k_B^A}{k}(E_A+b) - \beta\frac{{k_B^A}^2}{k^2}(E_A+b)\right]
\end{aligned} \tag{47}$$

Each of these four terms in the brackets can be dealt with separately in similar fashion:

$$\sum_{k_B^A+k_B^B+k_B^C=k} \frac{k!}{k_B^A!k_B^B!k_B^C!} q_{A|B}^{k_B^A} q_{B|B}^{k_B^B} q_{C|B}^{k_B^C} \left[\frac{k_B^A}{k}\right] = q_{A|B} \tag{48}$$

$$\sum_{k_B^A+k_B^B+k_B^C=k} \frac{k!}{k_B^A!k_B^B!k_B^C!} q_{A|B}^{k_B^A} q_{B|B}^{k_B^B} q_{C|B}^{k_B^C} \left[-\beta \frac{k_B^B k_B^A}{k^2}(E_B+d) \right]$$
$$= -\beta \frac{(E_B+d)}{k^2} k(k-1) q_{A|B} q_{B|B} \tag{49}$$

$$\sum_{k_B^A+k_B^B+k_B^C=k} \frac{k!}{k_B^A!k_B^B!k_B^C!} q_{A|B}^{k_B^A} q_{B|B}^{k_B^B} q_{C|B}^{k_B^C} \left[\beta \frac{k_B^A}{k}(E_A+b) \right] = \beta(E_A+b) q_{A|B} \tag{50}$$

$$\sum_{k_B^A+k_B^B+k_B^C=k} \frac{k!}{k_B^A!k_B^B!k_B^C!} q_{A|B}^{k_B^A} q_{B|B}^{k_B^B} q_{C|B}^{k_B^C} \left[-\beta \frac{{k_B^A}^2}{k^2}(E_A+b) \right]$$
$$= -\beta \frac{(E_A+b)}{k^2} k q_{A|B} [(k-1) q_{A|B} + 1] \tag{51}$$

Therefore,

$$\begin{aligned} W_{AB} = p_B \Bigg[& q_{A|B} + \beta \Big((E_A+b) q_{A|B} - \frac{E_A+b}{k} q_{A|B} \\ & - \frac{k-1}{k} q_{A|B} \big[(E_B+d) q_{B|B} + (E_A+b) q_{A|B} \big] \Big) \Bigg] + \mathcal{O}(\beta^2) \end{aligned} \tag{52}$$

Using the same techniques, we can simplify our expression for W_{BA}:

$$\begin{aligned} W_{BA} = p_A \Bigg[& q_{B|A} + \beta \Big((E_B+c) q_{B|A} - \frac{E_B+c}{k} q_{B|A} \\ & - \frac{k-1}{k} q_{B|A} \big[(E_B+c) q_{B|A} + (E_A+a) q_{A|A} \big] \Big) \Bigg] + \mathcal{O}(\beta^2) \end{aligned} \tag{53}$$

Note immediately that since $p_B q_{A|B} = p_A q_{B|A}$, the zero-th order terms of W_{AB} and W_{BA} are equal.

4.4 The ϕs:

The pair derivatives are non-zero, even when $\beta = 0$, so we will focus only on the zeroth order terms, because these will dominate the first-order terms when

β is small.

$$
\begin{aligned}
\phi_{AB}^{A} = p_B \cdot & \sum_{k_B^A + k_B^B + k_B^C = k} k_B^A \frac{k!}{k_B^A! k_B^B! k_B^C!} q_{A|B}^{k_B^A} q_{B|B}^{k_B^B} q_{C|B}^{k_B^C} \\
& \cdot \sum_{k_A^{A'} + k_A^{B'} + k_A^{C'} = k-1} \frac{(k-1)!}{k_A^{A'}! k_A^{B'}! k_A^{C'}!} q_{A|A}^{k_A^{A'}} q_{B|A}^{k_A^{B'}} q_{C|A}^{k_A^{C'}} \\
& \cdot \sum_{k_B^{A'} + k_B^{B'} + k_B^{C'} = k-1} \frac{(k-1)!}{k_B^{A'}! k_B^{B'}! k_B^{C'}!} q_{A|B}^{k_B^{A'}} q_{B|B}^{k_B^{B'}} q_{C|B}^{k_B^{C'}} \\
& \cdot \sum_{k_C^{A'} + k_C^{B'} + k_C^{C'} = k-1} \frac{(k-1)!}{k_C^{A'}! k_C^{B'}! k_C^{C'}!} q_{A|C}^{k_C^{A'}} q_{B|C}^{k_C^{B'}} q_{C|C}^{k_C^{C'}} \\
& \cdot \frac{k_B^A f_A(k_A^{A'}, k_A^{B'}+1, k_A^{C'})}{k_B^A f_A(k_A^{A'}, k_A^{B'}+1, k_A^{C'}) + k_B^B f_B(k_B^{A'}, k_B^{B'}+1, k_B^{C'}) + k_B^C f_C(k_C^{A'}, k_C^{B'}+1, k_C^{C'})}
\end{aligned}
\tag{54}
$$

The zeroth order terms are what is left when $\beta = 0$, or when we have neutral drift. In that case, $f_A = f_B = f_C = 1$, and most of the sums collapse to 1. We quickly get that

$$
\phi_{AB}^{A} = \frac{p_B}{k} \sum_{k_B^A + k_B^B + k_B^C = k} \frac{k!}{k_B^A! k_B^B! k_B^C!} q_{A|B}^{k_B^A} q_{B|B}^{k_B^B} q_{C|B}^{k_B^C} {k_B^A}^2 \tag{55}
$$

We relabel for notational convenience and readability when evaluating this sum. Let $X = k_B^A, Y = k_B^B, Z = k_B^C$. Then the sum is

$$
\begin{aligned}
& \sum_{X+Y+Z=k} \frac{k!}{X!Y!Z!} q_{A|B}^X q_{B|B}^Y q_{C|B}^Z X^2 \\
= & k q_{A|B} \sum_{(X-1)+Y+Z=k-1} \frac{(k-1)!}{(X-1)!Y!Z!} q_{A|B}^{X-1} q_{B|B}^Y q_{C|B}^Z (X) \\
= & k q_{A|B} \sum_{(X-1)+Y+Z=k-1} \frac{(k-1)!}{(X-1)!Y!Z!} q_{A|B}^{X-1} q_{B|B}^Y q_{C|B}^Z (X-1) \\
& + k q_{A|B} \sum_{(X-1)+Y+Z=k-1} \frac{(k-1)!}{(X-1)!Y!Z!} q_{A|B}^{X-1} q_{B|B}^Y q_{C|B}^Z \\
= & k q_{A|B} \Big((k-1) q_{A|B} \sum_{(X-2)+Y+Z=k-2} \frac{(k-2)!}{(X-2)!Y!Z!} q_{A|B}^{X-2} q_{B|B}^Y q_{C|B}^Z + 1 \Big) \\
= & k q_{A|B} \Big((k-1) q_{A|B} + 1 \Big)
\end{aligned}
\tag{56}
$$

Immediately, we get,

$$
\phi_{AB}^{A} = p_B q_{A|B} \Big((k-1) q_{A|B} + 1 \Big) + \mathcal{O}(\beta) \tag{57}
$$

The other ϕ terms are calculated in the same way. They are:

$$\phi_{BA}^{A} = p_A(k-1)q_{A|A}q_{B|A} + \mathcal{O}(\beta) \tag{58}$$

$$\phi_{BA}^{B} = p_A q_{B|A}\Big((k-1)q_{B|A} + 1\Big) + \mathcal{O}(\beta) \tag{59}$$

$$\phi_{AB}^{B} = p_B(k-1)q_{B|B}q_{A|B} + \mathcal{O}(\beta) \tag{60}$$

4.5 The Slow Manifold

With these simplified equations, we can solve the system. Consider the zero-th order terms, setting $\beta = 0$. $W_{AB} = W_{BA}$, so $\dot{p_A} = \dot{p_B} = \dot{p_C}$. Now we address $\dot{p_{AA}}$, $\dot{p_{BB}}$, and $\dot{p_{AB}}$:

With the above derivatives and (18), we get that

$$\dot{p_{AB}} = -\frac{1}{2}(\dot{p_{AA}} + \dot{p_{AA}}) \tag{61}$$

By substituting (57) and (58) into (41):

$$\begin{aligned}
\dot{p_{AA}} &= \frac{2}{k}\Big[\phi_{AB}^{A} - \phi_{BA}^{A}\Big] \\
&= \frac{2}{k}\Big[p_B q_{A|B}\Big((k-1)q_{A|B} + 1\Big) - p_A(k-1)q_{A|A}q_{B|A}\Big] \\
&= \frac{2}{k}\Big[p_B q_{A|B}q_{A|B}(k-1) - p_A q_{A|A}q_{B|A}(k-1) + p_B q_{B|A}\Big] \\
&= \frac{2}{k}\Big[\frac{p_{AB}^2}{p_B}(k-1) - \frac{p_{AA}p_{AB}}{p_A}(k-1) + p_{AB}\Big]
\end{aligned} \tag{62}$$

Similarly, with (59) and (60) in (42):

$$\begin{aligned}
\dot{p_{BB}} &= \frac{2}{k}\Big[\phi_{BA}^{B} - \phi_{AB}^{B}\Big] \\
&= \frac{2}{k}\Big[p_A q_{B|A}\Big((k-1)q_{B|A} + 1\Big) - p_B(k-1)q_{B|B}q_{A|B}\Big] \\
&= \frac{2}{k}\Big[p_A q_{B|A}q_{B|A}(k-1) - p_B q_{B|B}q_{A|B}(k-1) + p_A q_{B|A}\Big] \\
&= \frac{2}{k}\Big[\frac{p_{AB}^2}{p_A}(k-1) - \frac{p_{AB}p_{BB}}{p_B}(k-1) + p_{AB}\Big]
\end{aligned} \tag{63}$$

Now subtract (63) from (62):

$$\begin{aligned}
\dot{p_{AA}} - \dot{p_{BB}} =& \frac{2}{k}\Big[\frac{p_{AB}^2}{p_B}(k-1) - \frac{p_{AA}p_{AB}}{p_A}(k-1) + p_{AB}\Big] \\
&- \frac{2}{k}\Big[\frac{p_{AB}^2}{p_A}(k-1) - \frac{p_{AB}p_{BB}}{p_B}(k-1) + p_{AB}\Big] \\
=& \frac{2(k-1)}{k}\Big[\frac{p_{AB}^2}{p_B} - \frac{p_{AA}p_{AB}}{p_A} - \frac{p_{AB}^2}{p_A} + \frac{p_{AB}p_{BB}}{p_B}\Big]
\end{aligned} \tag{64}$$

When the system is initialized at $t = 0$, it is well-mixed and $p_{S_1 S_2}(0) = p_{S_1}(0)p_{S_2}(0)$ for all strategies S_1 and S_2. Thus, at $t = 0$, by equation (64), $\dot{p_{AA}} - \dot{p_{BB}} = 0$. And together with (61), we have

$$\dot{p_{AA}} = \dot{p_{BB}} = -\dot{p_{AB}} \tag{65}$$

In fact, this will hold for all time steps, because as long as it holds, it will continue to hold. A sketch of a formal proof is as follows: solve the system with Euler's method and take the limit as the discrete time step goes to zero. By the convergence of Euler's method, (65) holds for all t.

From this, (19) and (20) show that $\dot{p_{AC}} = \dot{p_{BC}} = 0$. Then,

$$\dot{q_{C|A}} = \frac{d}{dt}\frac{p_{AC}}{p_A} = \frac{\dot{p_{AC}} p_A - p_{AC}\dot{p_A}}{p_A^2} = 0 \tag{66}$$

Similarly, $\dot{q_{C|B}} = 0$. These results are expected because in neutral drift, the sanctioners do not give either strategy an advantage, so sanctioners will not naturally attract A players or repel B players.

Because β is very small, the zero-th order terms in $\dot{p}_{AA}$ and $\dot{p}_{BB}$ will go to zero much quicker than the first order terms in $\dot{p}_A$ and $\dot{p}_B$. Set $\dot{p}_{AA} = 0$:

$$\dot{p}_{AA} = \frac{2}{k}\Big[p_B q_{A|B}\Big((k-1)q_{A|B} + 1\Big) - p_A(k-1)q_{A|A}q_{B|A}\Big] = 0 \tag{67}$$

Rearranging and dividing by $\frac{2p_{AB}}{k}$ gives

$$(k-1)q_{A|B} + 1 = (k-1)q_{A|A} \tag{68}$$

Now use the identities $q_{A|B} = \frac{p_A}{p_B} q_{B|A}$ and $q_{B|A} = 1 - q_{A|A} - p_C$ and rearrange to get

$$q_{A|A} = p_A + \frac{p_B}{(k-1)(1-p_C)} \tag{69}$$

A similar procedure with $\dot{p}_{BB} = 0$ yields

$$q_{B|B} = p_B + \frac{p_A}{(k-1)(1-p_C)} \tag{70}$$

These conditions define the slow manifold, where the system changes slowly due to β being close to zero. The system may start as a well-mixed population, but it will very quickly approach a state where the above conditions hold, at least approximately. Notice that the slow manifold is one-dimensional; everything can be expressed in terms of p_A, because $p_B = 1 - p_C - p_A$, and p_C will be a constant.

4.6 Fixation Probabilities

Consider a system starting with $p_A(0) = p$ and a small time step Δt in which we assume one death-birth occurs. Renormalize with $p_{A\text{new}} = p_{A\text{old}}/(1 - p_C)$ and $p_{B\text{new}} = p_{B\text{old}}/(1 - p_C)$ so that p_A and p_B are between 0 and 1. Now p_A and p_B represent the proportion of individuals playing A or B out of all the

individuals that are capable of changing their strategy (the A and B players). There is a mean $m_A(p)$ and variance $v_A(p)$ of Δp_A for a single time step. We have

$$m_A(p) = E(\Delta p_A) = \frac{1}{N}[W_{AB} - W_{BA}] = [W_{AB} - W_{BA}]\Delta t \tag{71}$$

$$\begin{aligned} v_A(p) &= E(\Delta p_A^2) - E(\Delta p_A)^2 = E(\Delta p_A^2) + \mathcal{O}(\beta^2) \\ &\approx \frac{1}{N^2}[W_{AB} + W_{BA}] = \frac{1}{N}[W_{AB} + W_{BA}]\Delta t \end{aligned} \tag{72}$$

The relevant value will be $-\frac{2m_A(p)}{v_A(p)}$, which can be obtained by substituting in the constraints of the slow manifold: Equations (69) and (70). After substituting in the expressions for W_{AB} and W_{BA}, simplifying gets us:

$$-\frac{2m_A(p)}{v_A(p)} = \frac{\beta N}{k}(u_1 p + u_2) \tag{73}$$

where

$$u_1 = (a - b - c + d)(1 - k^2 - \frac{1+k}{p_C - 1})(1 - p_C) \tag{74}$$

$$u_2 = -a+b+c-d-ak+bk-bk^2+dk^2+(k-1)\big(c+(b-\alpha+\gamma)k-d(1+k)\big)p_C \tag{75}$$

According to diffusion theory, the fixation probability of A beginning with $p_A(0) = p$, denoted $\rho_A(p)$, satisfies the equation

$$m_A(p)\frac{d\rho_A(p)}{dp} + \frac{v_A(p)}{2}\frac{d^2\rho_A(p)}{dp^2} = 0 \tag{76}$$

This equation is separable and first order with respect to $\frac{d\rho_A(p)}{dp}$.

$$\ln\frac{d\rho_A(p)}{dp} = \int -\frac{2m_A(p)}{v_A(p)}dp \tag{77}$$

The low order terms are

$$\frac{d\rho_A(p)}{dp} = 1 + \frac{\beta N}{k}\Big(\frac{u_1}{2}p^2 + u_2 p\Big) + c_1 \tag{78}$$

c_1 is a constant of integration. Integrating once more gives

$$\rho_A(p) = p + \frac{\beta N}{k}\Big(\frac{u_1}{6}p^3 + \frac{u_2}{2}p^2\Big) + c_1 p + c_2 \tag{79}$$

Using the boundary conditions $\rho_A(0) = 0$ and $\rho_A(1) = 1$ to solve for the constants of integration, we get

$$\begin{aligned} \rho_A(p) =& p + \frac{\beta N}{k}\Big(\frac{u_1}{6}p^3 + \frac{u_2}{2}p^2 - (\frac{u_1}{6} + \frac{u_2}{2})p\Big) \\ =& p + \frac{\beta N p(1-p)}{6k}\Big(- 3u_2 - u_1(1+p)\Big) \end{aligned} \tag{80}$$

When $p \ll 1$, such as when $p = 1/N$ for invasion probabilities, (80) becomes

$$\rho_A(p) \approx p + \frac{\beta N p(1-p)}{6k}\Big(-3u_2 - u_1\Big) \tag{81}$$

We can use this work to calculate the fixation probability for the B strategy, as well. For a given p_A and p_B with $p_A + p_B = 1$, $m_B(p) = -m_A(1-p)$ and $v_B(p) = v_A(1-p)$. Therefore

$$\frac{-2m_B(p)}{v_B(p)} = \frac{2m_A(1-p)}{v_A(1-p)} = -\frac{\beta N}{k}\Big(u_1(1-p)+u_2\Big) = \frac{\beta N}{k}\Big(u_1 p-(u_1+u_2)\Big) \tag{82}$$

From this, as in (81),

$$\rho_B(p) = p+\frac{\beta N}{k}\Big(\frac{w_1}{6}p^3+\frac{w_2}{2}p^2-(\frac{w_1}{6}+\frac{w_2}{2})p\Big) \approx p+\frac{\beta N p(1-p)}{6k}\Big(-w_1-3w_2\Big) \tag{83}$$

with $w_1 = u_1$ and $w_2 = -(u_2 + u_1)$.

4.7 Sanctioner Accuracy

The adjustment to include a parameter λ to take into account inaccurate fact-checking is very simple. Recall that a sanctioner with accuracy $\lambda \in [0,1]$ gives benefit α to an A player and penalty γ to a B player with probability λ, and gives the opposite payoffs with probability $1-\lambda$. Therefore, the expected payoff an A player receives from a C player is $\lambda\alpha + (1-\lambda)\gamma$ and the expected payoff for a B player is $\lambda\gamma + (1-\lambda)\alpha$.

All the previous work with pair approximation, weak selection, and the diffusion approximation still hold, but we can replace the old expected payoffs of α and γ with the new expected payoffs $\lambda\alpha + (1-\lambda)\gamma$ and $\lambda\gamma + (1-\lambda)\alpha$, respectively. Conveniently, the substitution can be done at the very end, where α and γ appear as coefficients in Equation (75).
